# Is Deuterium Sequestering by Reactive Carbon Atoms an Important Mechanism to Reduce Deuterium Content in Biological Water?

**DOI:** 10.1096/fba.2025-00032

**Published:** 2025-05-14

**Authors:** Stephanie Seneff, Greg Nigh, Anthony M. Kyriakopoulos

**Affiliations:** ^1^ Computer Science and Artificial Intelligence Laboratory Massachusetts Institute of Technology Cambridge Massachusetts USA; ^2^ Greg Nigh LLC Westerly Rhode Island USA; ^3^ Department of Pharmacy University of Patras Rio‐Patras Greece; ^4^ Department of Research and Development Nasco AD Biotechnology Laboratory Piraeus Greece

**Keywords:** bilirubin, bis‐allylic carbon, carotenoids, deuterium, gut microbiome, histidine, imidazole propionate, lipid peroxidation, mitochondria, polyunsaturated fatty acids

## Abstract

Deuterium is a natural heavy isotope of hydrogen, having a neutron as well as a proton. Deuterium disrupts ATP synthesis in mitochondria, causing increased production of reactive oxygen species and reduced synthesis of ATP. Gut microbes likely play a significant role in providing deuterium depleted short chain fatty acids (SCFAs) to human colonocytes through hydrogen gas recycling. The production of deuterium depleted (deupleted) nutrients necessarily leaves behind deuterium enriched water, unless there is a process that can sequester deuterium in small molecules that are excreted through the feces. Here, we provide evidence that a small number of classes of uniquely structured carbon‐nitrogen rings and bis‐allylic carbon atoms in certain biologically active small molecules may play a crucial role in sequestering deuterium for export into feces or urine. Specifically, we have identified the imidazole ring present in histidine, histamine, and microbial derivatives of histidine, the tetraterpenoid lutein, bilirubin and the derivatives urobilinogen and stercobilinogen produced by gut microbes, and the bis‐allylic carbons in polyunsaturated fatty acids as likely candidates for sequestering deuterium and thereby reducing the deuterium levels in the water‐based medium. Normally, carbon atoms never exchange their bound protons with deuterons from the medium, but all the above classes of molecules are important exceptions to this rule, as has been shown experimentally.

## Graphical abstract

Deuterium, a heavy isotope of hydrogen, is universal in nature, but it disrupts the mitochondrial ATPase pumps. One mechanism that biological organisms may use to reduce deuterium levels in the mitochondria is to sequester deuterium bound to carbon atoms in a small set of organic molecules that have a unique configuration to support such trapping. These deuterium‐loaded molecules can be excreted in the feces or urine or stored in the extracellular domains.

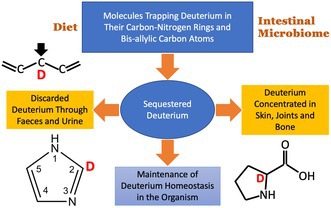

## 1 Introduction

Deuterium (^2^H), the nonradioactive heavy isotope of hydrogen, is a heretofore largely neglected factor in mitochondrial disease, although recent increased interest is rapidly changing the landscape. Mitochondrial dysfunction is a factor in a long list of debilitating chronic diseases, including Alzheimer's disease, Parkinson's disease, cardiovascular diseases, cancer, type 1 diabetes, multiple sclerosis, muscular dystrophy, metabolic syndrome, obesity, and various neurodegenerative disorders [1]. Deuterium is a natural element, found in seawater at 155 ppm. Studies on metabolic pathways clearly show that the enzymes involved in metabolizing organic molecules in the mitochondria result in the delivery of deuterium depleted (deupleted) protons to the mitochondrial intermembrane space [2]. In part, this feat is achieved through several dehydrogenase enzymes that bind nicotinamide adenine dinucleotide (NAD) and flavin adenine dinucleotide (FAD) and exploit proton tunneling to transfer a hydride ion from a carbon atom in the substrate to NAD^+^ to form NADH [3, 4]. Deuterons have been found to be 20 times less efficient at tunneling than protons [5], and this results in a high deuterium kinetic isotope effect (KIE). Mitochondrial NADH dehydrogenase, also known as Complex I multimeric enzyme complex, ultimately delivers protons to the intermembrane space, and these protons are highly unlikely to be ^2^H. It appears to be critical to maintain low deuterium levels in the intermembrane space to protect the F‐type ATPase pumps on the inner membrane from damage due to deuterium, which results in inefficient production of ATP and the release of excessive amounts of reactive oxygen species (ROS) [6].

A species of *Pseudomonas* produces hydrogen gas that is 80% depleted in deuterium from hydrogen extracted from simple organic molecules such as formate and glucose [7]. The *Pseudomonas* strain enzyme, hydrogenase, is similar to enzymes present in many of the gut microbes. H/D exchange mechanisms in high‐spin iron hydride complexes in hydrogenases such as the one expressed by *Desulfovibrio* species may play a role in the deupletion process [8]. This hydrogen gas is used by gut microbes to reduce carbon dioxide to organic carbon molecules, such as the short chain fatty acids, acetate, propionate, and butyrate [2]. Photoproduction of hydrogen gas by cyanobacteria also results in a similarly very low deuterium content in the hydrogen gas [9]. If the gut microbes are producing large quantities of deupleted nutrients, then it can be inferred that the water in the medium they operate in becomes enriched in deuterium. Therefore, Is there another process taking place in the gut to systematically remove deuterium atoms from water?

A small number of nitrogen‐carbon ring structures and other molecular configurations of carbon atoms appear to be unique in their ability to trap and retain deuterium. Once a molecule containing one of these special structures traps a deuterium atom, certain enzymatic reactions are greatly suppressed, because the enzyme that catalyzes the reaction has a very high deuterium KIE. Many of these molecules are excreted into the feces or the urine, because they are unable to be further metabolized. The unique properties of these organic molecules may play an essential role in maintaining low deuterium levels in biological water, something that is likely to be very important in the gut lumen. A small molecule with a single deuterium atom has a much higher concentration of deuterium than there is in seawater, where one out of every 6600 protons is a deuteron. Essentially, these molecules can be seen as finding and defusing deuterium “land mines.”

## 2 Vascular ATPase and Cancer

Vascular ATPase (v‐ATPase) is a class of enzymes that perform an opposite role compared to F‐type ATPase. Instead of producing ATP, they consume it, and they use the energy released to actively pump protons across a membrane, against an opposing pH gradient. This process can be used to acidify an intracellular vesicle or organelle, and/or to pump protons out of the cell, in order to create an electrochemical proton gradient to energize cellular functions such as nutrient import [10].

The class is named after the yeast v‐ATPase, the first member identified. Studies on yeast cultured in heavy water have revealed an astonishing deuterium KIE for the transport of protons/deuterons across a membrane, driven by v‐ATPase pumps. The authors wrote in their paper: “It thus appears that the binding site for protons (or hydronium ions) to be transported does not accept deuterons (or deuteronium ions) with equal ease or perhaps not at all.” [11] One conclusion one can draw is that steady increased activity of the ATPase pumps will ultimately result in deuterium enrichment in the cellular water and deuterium depletion in the extracellular space.

A study published in 1988 investigated what happens to mouse and monkey fibroblast cells if they are endowed with the yeast v‐ATPase enzyme. They achieved this feat by inserting the yeast v‐ATPase gene into a mammalian expression plasmid, under the control of an SV40 promoter. They found that this gene had oncogenic properties, transforming the cells into tumorigenic cells that were able to produce tumors in exposed mice. Furthermore, they confirmed that the intracellular pH of the v‐ATPase transfected cells was higher than that of cells lacking the enzyme, as expected [12]. A case has been made, based in part on this observed phenomenon, that cancer cells sequester deuterium in order to reduce the deuterium burden in the extracellular space, helping the resident immune cells restore health to their mitochondria, ultimately empowering them to attack and clear the cancer [13].

## 3 Hydrogen/Deuterium Exchange: A Brief History

Hydrogen/deuterium exchange (HDX) is a chemical process that routinely takes place in organic molecules immersed in a water‐based solvent. Oxygen, nitrogen, sulfur, and carbon, the four primary atoms besides hydrogen in organic molecules, have differing degrees of exchange rates with protons/deuterons in the water. Generally, the HD exchange rate follows the rule of oxygen > nitrogen > sulfur > carbon.

Labile hydrogens in the backbone and side‐chain functional groups of proteins undergo exchange with protons in the solvent within a few minutes. The rate of the reaction is strongly dependent on pH, with a greater exchange rate under basic conditions. Hydrogens in carboxyl groups exchange more readily than those in amide groups. Steric hindrance can drastically alter the rate [14].

Under usual circumstances, carbon atoms do not exchange at all, but there are exceptions, and they may be highly significant. It is also likely that exchange happens much more readily through enzymatic action, and, in fact, the class of enzymes called isomerases may play an important role in biology in stripping deuterium from organic molecules. There is an apparent futility in isomerase reactions, but this is deceptive because most of them result in redistributing deuterium between the two isomers and in exchange with the water medium.

A good example is triosephosphate isomerase, a core enzyme of the glycolysis pathway. During glycolysis, the six‐carbon glucose molecule is phosphorylated and split into two phosphorylated 3‐carbon sugars, dihydroxyacetone phosphate (DHAP) and glyceraldehyde‐3‐phosphate (G3P), which are isomers of each other. At equilibrium, 95% of the triosephosphates are DHAP. Yet only G3P continues along the glycolysis pathway, with the next step being a dehydrogenase that rapidly converts NAD^+^ to NADH. The isomerase forms an enzyme‐enediol intermediate, and any deuterium present at that time will most likely exchange with a proton in the medium that displaces the deuteron, such that a proton ends up on the product [15]. Repeated cyclic activity of the isomerase eventually completely scrubs deuterium from the carbon atom in G3P that ultimately delivers a proton to NAD^+^ via the dehydrogenase. As a result, the hydrogen atom of the NADH produced by the dehydrogenase is severely deupleted. Experiments on deuterium tracing have shown that, if C_1_ of glucose is doped with deuterium that is then traced through metabolic pathways, it gets lost to the water during the activity of triose phosphate isomerase [16].

The sulfhydryl group of cysteine residues resists exchange with water hydrogens [17]. and this may be an important consideration in the activity of protein disulfide isomerase, which assists protein folding in the endoplasmic reticulum [18]. Glutathione reductase restores protons to the ‐SH groups of cysteine in glutathione sourced from NADPH (derived from mitochondrial NADH), so these protons should be deupleted [2, 19]. In turn, glutathione peroxidase delivers these protons to hydrogen peroxide to produce two molecules of deuterium‐depleted water, helping to maintain low deuterium content in the mitochondrial water.

The α carbon (α‐C) of amino acids is the core carbon atom that is attached to both the amino group and the carboxyl group that links up with other amino acids to form the peptide chain. Usually, the α‐C is resistant to HDX, but modifications to the amino acid can increase its ability to pick up a deuterium atom from the medium under basic conditions. Furthermore, if the deuterated molecule is returned to acidic conditions, the deuterons remain attached and do not back exchange with protons in the water [20, 21]. This is in contrast to deuterium atoms bound to nitrogen and oxygen, which freely exchange with the water under all conditions, which makes them ineffective at sequestering deuterium.

N‐substituted glycine peptoids (glycine oligomers) that possess deuteron‐substituted α‐carbon atoms can be created by simply immersing them in a deuterium‐rich basic medium. The addition of a methyl group attached to the nitrogen atom in a glycine residue, yielding sarcosine, greatly increases the ability of the α‐carbon to pick up deuterium. These deuterated peptoids resist degradation and do not undergo back exchange under acidic conditions [22, 23]. The unique ability of carbon atoms to trap and sequester deuterium under special circumstances may be a significant factor in maintaining reduced deuterium levels in the gut lumen and in the circulation.

## 4 Lutein and Other Carotenoids

Lutein is a member of the biologically active carotenoid family of molecules, well known for their antioxidant effects. It is a natural molecule produced by many plants. Human cells are unable to synthesize lutein, so it can only be obtained from the diet. Lutein has been shown to have promising beneficial effects to protect from eye diseases, cardiac complications, microbial infections, skin irritation, and bone decay [24].

**FIGURE 1:**
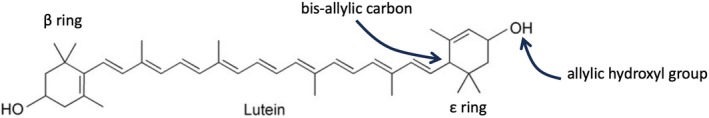
Structure of the lutein molecule, highlighting the bis‐allylic carbon atom and the allylic hydroxyl group in the ε ring on the right‐hand side of the molecule.

The freshwater algae Chlorella can be exploited in the laboratory to produce lutein that is highly enriched in deuterium. It was shown that a 58% uptake of deuterium substituting for protons bound to carbon atoms in lutein could be achieved by growing Chlorella in heavy water. Deuterated lutein has been used experimentally to trace lutein's pathways through the body. Lutein's molecular structure is shown in Figure 1. It has two rings, one on each end of a long, highly desaturated, 18‐carbon chain (alternating double bonds and single bonds down the chain). There are four methyl groups branching off certain carbon atoms in the chain. The LHS ring is known as the α ring, and the RHS ring is called the ε ring. The ε ring is of particular interest, because it contains a bis‐allylic carbon atom (a carbon atom single‐bonded to neighbors on both the left and the right that are both double‐bonded to their other neighbor in the chain), as shown in Figure 1. It also contains an allylic hydroxyl group, which can be removed enzymatically in the acidic environment of the stomach to yield anhydrolutein and a water molecule. While much of the lutein was converted in an acidic environment to anhydrolutein, none of the deuterated lutein molecules were dehydrated, likely due to a high deuterium KIE for removal of the deuterons under acid conditions [25]. This implies that the water that is produced by dehydrating lutein is deupleted but also that the ring can potentially trap and sequester deuterium.

These unusual features of lutein are elegantly designed to be able to (1) enzymatically yield deupleted water as a dehydration reaction product, and (2) trap and sequester deuterium atoms from the water. The key point is that, once they acquire a deuterium atom attached to a specific carbon atom, the reaction no longer takes place, due to a high deuterium KIE for the back exchange reaction under acidic conditions.

Another carotenoid, *β*‐carotene, can also become highly deuterated with 60%–65% of the hydrogen atoms becoming deuterated [25]. It may be that all the carotenoids share a common feature of facilitating deuterium removal from water in the gut lumen in this way.

## 5 Hemoglobin Metabolism and Bilirubin Cycling

Red blood cells (RBCs) are the most common cell type in the body. Remarkably, because of the absence of a nucleus, they only live on average for about 4 months, so aged RBCs must be constantly removed from circulation by the spleen, and then erythropoiesis supports their regeneration in the bone marrow. Equilibration of production and destruction of RBCs maintains a relatively constant concentration of ∼5 million cells per μL of blood. Macrophages in the red pulp in the spleen recognize senescent RBCs and remove them from circulation through phagocytosis [26].

By far the most common protein in RBCs is hemoglobin, which utilizes iron bound to heme to carry oxygen. Heme is mainly broken down in the spleen, and is resynthesized in the bone marrow and the liver. Heme oxygenase‐1 (HO‐1) is highly active in the spleen, and it converts heme derived from RBCs to biliverdin, releasing carbon monoxide (CO) and free iron (Fe^2+^), and producing three molecules of water by reducing oxygen, with each biliverdin molecule produced [27]:

(1) $$\text{heme}+{\text{3O}}_{2}➔\text{biliverdin}+{\text{3H}}_{2}O+{\mathrm{Fe}}^{2+}+\mathrm{CO}+H^{+}$$

NADPH‐cytochrome P450 reductase provides the electrons for the catalytic turnover of HO‐1. This enzyme restores the bound NADPH from NADP^+^ after the reaction is completed. It is a membrane‐bound flavoprotein that uses both FAD and flavin mononucleotide (FMN) as cofactors [28]. Thus, the protons provided by the reductase will be deupleted through proton tunneling.

The metabolism of biliverdin involves several steps, mostly carried out by the gut microbes. Biliverdin is water soluble, but its metabolites are not. The human protein biliverdin reductase, expressed primarily in the spleen and liver, reduces biliverdin to bilirubin. The spleen ships both biliverdin and bilirubin, via the hepatic portal vein, to the liver. The liver processes these molecules and ships them on to the gut in the bile acids. The gut microbes produce many metabolites derived originally from biliverdin [29].

Bilirubin has become recognized as a potent antioxidant, and elevated levels in the vasculature are generally viewed as being beneficial against many chronic conditions, including inflammation, diabetes, cardiovascular disease, metabolic syndrome, obesity, Crohn's disease, and chronic liver disease [29, 30]. Bilirubin is insoluble in water. As an open chain tetrapyrrole, it has an extended conjugated double‐bond system, allowing it to be able to act as an antioxidant by donating protons to hydrogen peroxide to produce two water molecules. Bilirubin is conjugated in the liver to glucuronic acid and secreted via the biliary tract into the upper intestine. Unconjugated bilirubin that makes it past the liver into the general circulation cannot be secreted from the body. It is carried in the blood bound to serum albumin, and it is a potent antioxidant [30].

## 6 Gut Microbial Processing Beyond Bilirubin: Bis‐Allylic Carbon

The enzyme synthesized by the gut microbes that further reduces bilirubin to other degradation products had long remained elusive, but a recent paper has finally identified the enzyme and characterized its enzymatic activity [31]. This enzyme, bilirubin reductase (BilR) has a flavodoxin‐like domain and an NADP(H)‐binding domain that facilitate electron transfer. It is primarily synthesized by *Firmicutes* bacteria. The reduction reaction involves a hydride transfer to the carbon–carbon double bond of bilirubin from a flavin cofactor. Due to proton tunneling phenomena in flavoproteins, one can confidently say that the two protons that are added to bilirubin are deupleted. Remarkably, there is a whole sequence of increasingly reduced products of bilirubin produced by the gut microbes, as shown in Table 1. All these molecules have an identical formula except for the addition of protons with each reduction step. Biliverdin has 34 protons, whereas stercobilinogen, the most reduced metabolite, has 48, an addition of 14 protons in total. It is likely that bilirubin reductase is the microbial enzyme that catalyzes all the reactions that ultimately produce stercobilinogen.

**TABLE 1:** Chemical formulas of biliverdin, bilirubin, and all the subsequent microbial metabolites of bilirubin. All the molecules have the same number of carbon, nitrogen and oxygen atoms, but with increasing numbers of hydrogen atoms with each step of reduction by microbes.

Molecule	Chemical Formula	# Hydrogens
Biliverdin	C_33_H_34_N_4_O_6_	34
Bilirubin	C_33_H_36_N_4_O_6_	36
Mesobilirubin	C_33_H_40_N_4_O_6_	40
Urobilin	C_33_H_42_N_4_O_6_	42
Urobilinogen	C_33_H_44_N_4_O_6_	44
Stercobilin	C_33_H_46_N_4_O_6_	46
Stercobilinogen	C_33_H_48_N_4_O_6_	48

**FIGURE 2:**
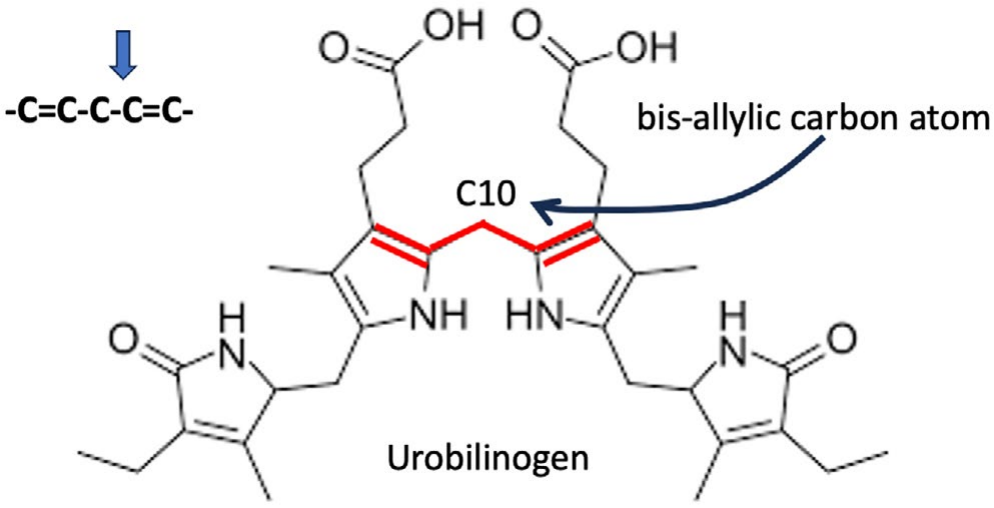
Urobilinogen's C10 carbon atom is an example of a bis‐allylic carbon atom: –C=C–C*–C=C–, capable of trapping and sequestering deuterium.

Like bilirubin, urobilinogen has a radical scavenging capability that protects lipids from peroxidation, as demonstrated experimentally in a study published in 2006 [32]. Its scavenging ability was found to be superior to that of other known antioxidants, including α‐tocopherol, bilirubin, and β‐carotene. This study is notable because the authors exposed urobilinogen to high concentrations of deuterated methanol, and the oxygen atoms and nitrogen atoms all replaced their protons with deuterons, as expected. However, surprisingly, the two protons attached to the bis‐allylic C_10_ carbon atom of urobilinogen were also swapped out for deuterons (see Figure 2). This suggests that urobilinogen, and probably other powerful antioxidants, serve a powerful and heretofore overlooked role in sequestering and removing deuterium atoms from the water. The stercobilinogen molecule, the final product of bacterial metabolism of bilirubin, also contains a bis‐allylic carbon atom, and it too probably has a similar property of being able to trap deuterium on its C_10_ carbon atom. These reaction products of heme produced by the microbes are ultimately excreted in the feces, giving them their characteristic dark color, and in the urine, where they are responsible for its yellow tinge.

## 7 The Significance of Bis‐Allylic Carbon Atoms in Polyunsaturated Fatty Acids

### 7.1 Lipid Peroxidation of Polyunsaturated Fatty Acids

**FIGURE 3:**
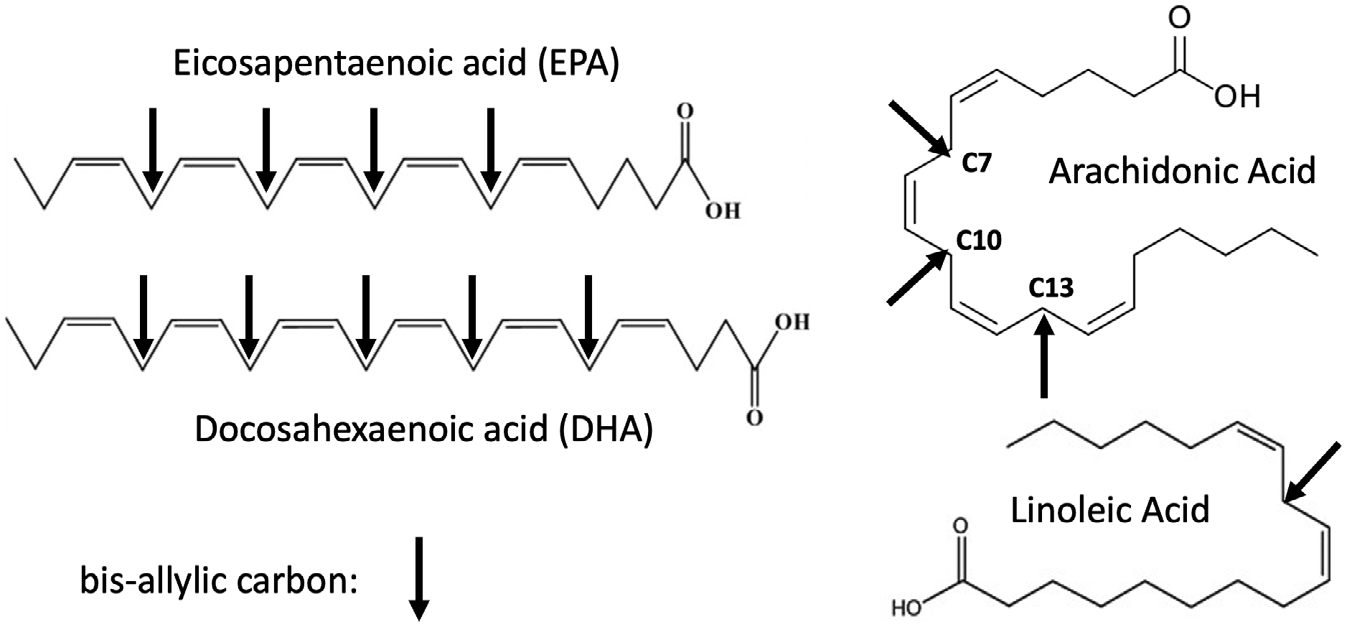
Examples of bis‐allylic carbon atoms in four different polyunsaturated fatty acids (PUFAs).

Polyunsaturated fatty acids (PUFAs) are a large class of lipids that populate the membranes of human cells, with an essential role for maintaining membrane stability by influencing the fluidity and flexibility of the plasma membrane. All the main lipid components of plasma membranes have bis‐allylic carbon atoms. While the *ω*‐6 fatty acid linoleic acid contains only one bis‐allylic carbon, its derivative, arachidonic acid, contains three, at positions C_7_, C_10_, and C_13_. The *ω*‐3 fatty acids that are prevalent in neuronal membranes, eicosapentaenoic acid (EPA) and docosahexaenoic acid (DHA), contain even more bis‐allylic carbons—four for EPA and five for DHA [33]. Figure 3 illustrates bis‐allylic carbon atoms in PUFAs.

A lipid peroxidation chain reaction is a self‐propagating process whereby a free radical attacks a PUFA at a bis‐allylic carbon, leading to the formation of a lipid radical which can then react with oxygen to create a peroxyl radical. This radical in turn can attack another fatty acid, continuing the chain reaction and causing significant damage to the membrane [34]. Lipid peroxidation has been implicated in many disease states, including atherosclerosis, inflammatory bowel disease, asthma, Parkinson's disease, kidney damage, and preeclampsia, among others [35].

Despite their ability to cause extensive cellular damage through a reaction cascade, dietary PUFAs are generally considered to be beneficial to health, which seems contradictory [36]. Lipid peroxidation is therefore referred to as a “double‐edged sword.” Cycloxygenases (COX) and lipoxygenases (LOX) are upregulated in many cancers [37, 38]. Their expression launches a reaction cascade that yields inflammatory signaling products called eicosanoids, which are derived from PUFAs, particularly arachidonic acid, following cleavage by phospholipase A2 [39]. Prolonged unresolved inflammation can cause tissue damage due to oxidative stress, especially when antioxidant defenses are insufficient. “Eicosanoids” is a broad term that encompasses leukotrienes, prostaglandins, prostacyclins, and thromboxanes, powerful signaling molecules that can launch an inflammatory cascade [40]. The rate of lipid peroxidation increases exponentially with the number of bis‐allylic carbons and is completely independent of the total length of the chain [41].

The oxidation of PUFAs is initiated by hydrogen abstraction at the bis‐allylic positions, yielding a hydroperoxyl radical that initiates a chain reaction that can run multiple cycles before quenching [42]. The capture of a deuterium atom from the medium at a bis‐allylic carbon may be the mechanism by which quenching eventually takes place. Deuteration of only a small fraction of PUFAs in a membrane can have a dramatic effect in protecting from the reaction cascade upon lipid peroxidation, far out of proportion to their concentration [43]. The bis‐allylic methylene positions in PUFAs have a weaker hydrogen bond dissociation energy (75–80 kcal/mol) compared to monoallylic hydrogen (88 kcal/mol), and an alkyl C‐H bond (where the carbon neighbors have no double bonds) is considerably stronger, with a 101 kcal/mol dissociation bond energy [9]. Thus, the bis‐allylic carbons more readily exchange their protons with deuterons from the water. Furthermore, PUFAs that are deuterated at the bis‐allylic carbon positions have a remarkable ability to quench lipid peroxidation chain propagation [42].

### 7.2 Can Deuterated PUFAs Be Therapeutic in the Plasma Membrane?

The pharmaceutical industry is exploring the possibility of providing deuterated PUFAs as nutritional supplements to help protect from lipid peroxidation damage. The rate limiting step in PUFA autoxidation is a hydrogen abstraction from a bis‐allylic methylene group. A study demonstrated that deuterated PUFAs can extend the lifespan of the worm, *C. elegans* [44]. These authors wrote in the abstract: “Chemically reinforced essential fatty acids (FAs) promise to fight numerous age‐related diseases including Alzheimer's, Friedreich's ataxia and other neurological conditions. The reinforcement is achieved by substituting the atoms of hydrogen at the bis‐allylic methylene of these essential FAs with the isotope deuterium” [44].

Lipid peroxidation is a major contributor to macular degeneration. An experiment involving administering DHA deuterated at the bis‐allylic carbon positions to mice showed that, after 77 days of administration, deuterated PUFAs appeared at high concentrations (above 90% of the total PUFAs) in all tissues except the central nervous system, where it only reached 75% to 80% penetration. They showed that it crosses the blood‐retina barrier and protects from lipid peroxidation [45].

Physiological lipid metabolism, which includes both lipid oxidation and peroxidation, must be viewed as a normal signaling process for all mammals, including humans. The introduction and implementation of deuterated lipids to inhibit lipid peroxidation may not be a net benefit for the maintenance of health. ROS and lipid peroxidation provide a profound signaling mechanism for cells to induce apoptosis and autophagy, which are normal cellular processes [46]. The study of R. Volinsky et al. has specifically shown that oxidized phosphatidylcholines in liposomes provide a direct non‐enzymatic signaling mechanism for apoptosis by altering the biophysical properties of lipid membranes and inducing transbilayer diffusion (a phospholipid flip‐flop) of membrane lipids [47].

Overall, under physiological conditions, the acceleration of transbilayer diffusion is a normal process. The loss of control of lipid asymmetry has profound effects on autophagy dysregulation, apoptosis, and cancer [48]. The intactness of lamellar repeat spacing and bilayer thickness of lipid membranes is necessary to maintain the asymmetry of lipid bilayers by, for example, maintaining the symmetrical distribution of cholesterol in these bilayers [49]. Specific studies on deuteration of lipids and their effect on membrane structure have shown that deuteration causes significant changes in the membrane lamellar repeat spacing and bilayer thickness. This is due to the lowering of the gel‐fluid phase transition temperature over that of protonated lipids. The deuteration of lipid chains causes a reduction of both lamellar repeat spacing and bilayer thickness. In contrast, the deuteration of lipid headgroups causes an increase in both of these lipid membrane features [50].

Furthermore, the normal process of lipid peroxidation by ROS provides the right signaling for Hypoxia‐induced gene domain protein‐1a (Higd1a) expression, an important protein that maintains the mitochondrial transmembrane potential and regulates apoptosis. The increased expression of Higd1A by peroxidation of lipids is coupled with the upregulation of Hypoxia Inducible Factor 1 (HIF‐1) and Peroxisome Proliferator‐activated receptor Gamma Coactivator 1‐alpha (PGC‐1α) proteins [51]. Apart from the mitochondrial protection offered by the lipid peroxidation of the Higd1A‐HIF‐1‐/PGC‐1α activation axis, PGC‐1α is a highly important protein for mitochondrial biogenesis and prevention of diseases such as diabetes mellitus and neuronal degeneration [52]. These conditions can be detrimentally impacted by the introduction of chemically modified deuterated lipids in lipid membranes. Recent evidence suggests that the maintenance of the normal hydrogen to deuterium ratio in the organism, and therefore in cells, is essential for the balance of mitochondrial oxidation and normal growth rate signaling (for review see Ref. [53]).

### 7.3 A Role for Deuterium in the Resolution of Inflammation

While the products of COX are strictly pro‐inflammatory, LOX produces both pro‐inflammatory and anti‐inflammatory products, and there is a time course over which an inflammatory response initially induces inflammation via inflammatory metabolites. After some time has passed, products that restore tissue homeostasis emerge, such as resolvins, protectins, and lipoxins [54, 55]. The *ω*‐3 fatty acids DHA and EPA can yield resolvins and protectins, whereas lipoxins evolve primarily from arachidonic acid.

DHA is the most abundant PUFA in the photoreceptor membranes in the eyes. DHA oxidation is central to the pathogenesis of iron‐induced retinal degeneration. Oxidation of DHA leads to carboxyethylpyrrole (CEP) adducts, which are associated with macular degeneration. In a mouse model of macular degeneration, providing the mice with dietary DHA deuterated at the bis‐allylic carbon atoms protected them from iron‐induced retinal degeneration by reducing lipid peroxidation [56].

Lipoxin B4 (LXB4), a product of the lipoxygenation of arachidonic acid by 15‐LOX, has been found to promote the resolution of inflammation in the respiratory tract of mice [57]. LXB4 inhibited mast cell degranulation and significantly decreased airway inflammation and mucus metaplasia. More generally, lipoxins both inhibit pro‐inflammatory factors and stimulate anti‐inflammatory factors, and they have been shown to inhibit neutrophil activation and promote phagocytosis of apoptotic neutrophils by macrophages [58].

Interestingly, it has been discovered that deuteration of the C_10_ bis‐allylic carbon atom in arachidonic acid causes a significant increase in the production of LXB4 by macrophages, likely because deuteration suppresses COX activity due to a large deuterium KIE. This redirects the arachidonic acid molecule towards LOX metabolism, which produces LXB4 [59]. The experiment that led to this discovery involved a careful study of the effects of deuteration on each of the three bis‐allylic carbons in arachidonic acid—singly and in combination—on the activity of COX and LOX enzymes. The authors were surprised to find that C_10_ deuteration had such a profound effect on activity. They proposed a mechanism that involves interference with the arachidonic acid cyclization step that takes place in the COX pathway, resulting in a shunting to the LOX pathway instead [59]. The mechanism of cyclization may involve a carbocation intermediate centered at C_10_, and deuterium substitution interferes with this step [60].

Remarkably, prostaglandin F2α (PGF2α), a product of COX metabolism, showed an astonishing deuterium KIE effect when both protons attached to C_10_ were deuterated, calculated at 270. The authors wrote: “it is possible that the large PKIE [physiological KIE] is due to inhibited keto‐enol tautomerization caused by deuterium present at C_10_.” [59] When both C_10_ and C_13_ were fully deuterated, virtually no detectable prostaglandins or thromboxanes, products of COX oxidation, were produced by activated macrophages.

Aldehyde dehydrogenase 2 (Aldh2) null mice are an established model of cognitive impairment due to oxidative stress. When these mice were fed PUFAs for 18 weeks that were deuterated at bis‐allylic carbon atoms, there was a markedly decreased production of F2‐isoprostanes and PGF2α compared to unenriched PUFAs. D‐PUFAs also improved their performance on memory and cognitive tests [61]. As we have seen, PGF2α is severely suppressed when the C_10_ carbon in arachidonic acid is deuterated. Isoprostanes are reaction products of the peroxidation of arachidonic acid that occur spontaneously [62]. The fact that deuterated lipids reduce their levels is a clear indicator that they are very effective at quenching the reaction cascade.

These results suggest that the inflammatory response may be a strategy to help decrease deuterium levels in the extracellular space. The initiation step in lipid peroxidation involves the production of a fatty acid radical when a hydroxyl radical attacks a hydrogen atom in the lipid to form water and a fatty acid radical. This unstable radical quickly reacts with molecular oxygen in the propagation step to form a peroxyl‐fatty acid radical. The water molecules that are produced during the cascade reaction will be deupleted, because lipids tend to be low in deuterium. However, the attack by the hydroxyl radical provides an opportunity to replace a proton bound to a bis‐allylic carbon, particularly C_10_ in arachidonic acid, with a deuterium atom from the water. The beauty of this system is that, once deuterium displaces the hydrogen atom, the enzyme's ability to catalyze the reaction is severely decreased. The lipids that acquire deuterium in this way are taken out of the game, but, more than that, they quench the fire.

In animal models, researchers are exploring the possibility of providing deuterated lipids as a nutrient source in the hopes of alleviating Alzheimer's symptoms. A study using a mouse model of Alzheimer's disease, involving mice that had mutated forms of both human amyloid precursor protein (APP) and the presenilin 1 gene, found that supplementing these mice with deuterated PUFAs for 5 months led to high levels of deuterated PUFA incorporation into arachidonic acid and docosahexaenoic acid in the brain, along with a reduction in the lipid peroxidation products, F2 isoprostanes and neuroprostanes. However, disappointingly, there was no observed improvement in cognitive defects [63]. It is possible that the initial inflammatory state is an important component of the lipid peroxidation reaction, because it both produces deuterium depleted water and traps deuterium at C_10_. If deuterium is already present in many of the lipid molecules, the process of lowering deuterium levels in the medium is aborted.

### 7.4 A Novel Proposed Role for Cardiolipin

Cardiolipin is a unique cone‐shaped lipid that is synthesized at the inner membrane of the mitochondrial intermembrane space. The main lipid present in cardiolipin in the heart muscle is linoleic acid, which contains a single bis‐allylic carbon atom [64]. In neurons, however, cardiolipin is strikingly enriched in long‐chain PUFAs, particularly DHA, which has five bis‐allylic carbon atoms [65]. Exogenous arachidonic acid, EPA, and DHA are readily incorporated into mitochondrial cardiolipin [66]. Cardiolipin localizes to the inner membrane where it facilitates the organization of the enzymes involved in electron transport and ATP production into supercomplexes [67]. Cardiolipin is tightly associated with ATPase pumps [64]. It has been hypothesized that a role it plays is to buffer protons as they traverse the pumps [68]. But a distinct possibility is that the bis‐allylic carbon atoms in cardiolipin trap and sequester deuterium atoms as they pass through the pumps. If this is true, it would be of great benefit to the cell, both because deuterons are kept away from the ATPase motor and because the deuterium‐loaded PUFAs would be able to halt a chain reaction lipid peroxidation cascade.

Cardiolipin's enrichment in PUFAs makes it highly vulnerable to peroxidation, and, in the presence of reactive oxygen, cytochrome c transforms into a cardiolipin peroxidase [69]. Under extreme oxidative stress conditions, oxidized cardiolipin binds to cytochrome c and migrates to the outer membrane, where it facilitates cytochrome c release and the initiation of an apoptotic cascade reaction, essentially targeting the severely damaged cell for destruction [70, 71]. Interestingly, programmed apoptotic signaling in neurons during the perinatal period leads to massive neuronal cell death and remodeling, via cytochrome c release. Already by 4 months of age, mouse neuronal cardiolipin molecules have acquired remarkably more arachidonic acid (three bis‐allylic carbon atoms) and DHA (five bis‐allylic carbon atoms) than cardiolipin from other tissues, and this increase is sustained into adulthood [65]. While this is highly speculative, it may be that the massive neuronal die‐off at birth releases an abundant supply of deuterated PUFAs that then populate the remaining neurons to provide long‐term protection against peroxidation chain reactions.

## 8 Histidine, Histamine, Histamine Metabolites, and the Imidazole Ring

### 8.1 C_2_ In the Imidazole Ring Traps Deuterium

Molecules containing an imidazole ring are another class that we suspect is highly amenable to deuterium trapping on a carbon atom, specifically the C_2_ carbon that is sandwiched between two nitrogen atoms in the ring. Histidine is the only amino acid with a pKa value close to physiological pH. This means that its charge can readily change with small pH fluctuations.

When dendrimers are immersed in D_2_O, protons are displaced by deuterons on all the oxygen and nitrogen atoms, but C_2_ of histidine is the only carbon atom that gets its proton replaced by a deuteron. Importantly, when the molecule is then immersed in H_2_O at an acidic pH, the deuterium rapidly disappears from all the nitrogen and oxygen atoms, but it remains in place on the C_2_ atom in the imidazole rings of histidine residues [72]. This suggests that imidazole rings may be able to be exploited to trap deuterium permanently.

Deuteration experiments involving the imidazole ring have become a powerful tool for studying peptide structure. Researchers have been able to learn a great deal about the structure of proteins through a technique called histidine hydrogen‐deuterium exchange mass spectrometry (His‐HDX‐MS), which combines mass spectrometry with HDX. This technique can determine both the pKa values of histidine imidazole groups and quantify their solvent accessibility [73].

Because the pKa value of imidazole NH groups is sensitive to adjacent charged groups, it is an indicator of the electrostatic environment of the ring. Cebo et al. showed in 2014 that the DHX reaction of the C_2_ proton in the ring is sensitive to both metal ion complexation and accessibility to the bulk solvent. N‐phosphorylation greatly reduces the ability of histidine to pick up deuterium on C_2_. By evaluating several different histidine‐containing peptides, these authors verified that the k values for HDX differed by about two orders of magnitude between phosphorylated and unphosphorylated histidine residues [74].

Imidazole rings have very different pKa values depending upon the neighboring charged groups. A seminal study published in 2012 systematically evaluated the rate of HDX for various small organic molecules containing an imidazole ring as a function of pH. These authors determined plots of the rate of HDX as a function of pH for four metabolites of histidine: histamine (pKa = 5.42), N‐acetyl‐L‐histidine methylamide (pKa = 6.35), N‐acetyl‐L‐histidine (pKa = 7.38), and imidazole propionic acid (ImP; pKa = 7.77). For all four of these molecules, the curve has a sigmoidal shape, with the maximum rate of exchange corresponding to the highest pH value, and the midpoint of the curve occurring at the pKa, where there is a steep rise in the k for HDX. ImP, having the highest pKa, also had the highest maximum rate of HDX. There was a near‐perfect linear relationship between the log of k_max_ and the pKa [75]. In other words, the rate of exchange goes up exponentially with the pKa. Most notably, ImP is the most effective molecule in the group for trapping deuterium on C_2_.

By quantifying the relationship between pKa and HDX, the authors were able to measure the solvent accessibility of histidine imidazole groups in proteins, and they defined a “protection factor” reflecting the ratio of the observed HDX rate to the expected rate with full solvent accessibility [75]. This result makes HDX in histidine residues a valuable tool for assisting in determining protein structure.

### 8.2 Metabolism of Histidine by Intestinal Flora and Mast Cells

A collection of diseases, from cancer to autoimmune and inflammatory disorders of the gastrointestinal system, has been associated with the distribution of microbes colonizing the digestive tract. During food intake, the amino acid L‐histidine, contained in food proteins, can be readily converted to histamine by histidine decarboxylase (HDC), expressed by numerous bacterial species in the gut [76]. Indeed, the histidine‐to‐histamine production by the microbial flora of the intestines and subsequent H2 receptor stimulation has gained an increasing amount of medical attention. Probiotic histamine‐producing bacteria can beneficially impact pathological conditions, including cancer, inflammatory bowel disease, colitis, irritable bowel syndrome, and beyond [77].

Besides histamine produced by gut microbes from dietary histidine, histamine is also readily produced by certain cells of the human immune defense system, especially the mast cells [78]. Mast cell activation syndrome is a condition where mast cells are overactive and release excessive amounts of histamine, causing a variety of symptoms, including swelling, diarrhea, hives, and severe allergic reactions [79]. There are several degranulating agents that can potentiate the release of histamine from mast cells. It has been shown that D_2_O in the culture medium not only increases the ability of these agents to induce degranulation, but also, at sufficiently high concentration, induces degranulation and histamine release on its own [80].

Apart from being metabolized to histamine by HDC‐expressing bacteria and mast cells in the gut, histidine is also readily converted to another metabolite, namely imidazole propionate (ImP) by the gut microflora, via the enzyme urocanate reductase (UrdR) [81]. This metabolite also has a wide range of powerful effects on host cells. The work of A Koh et al. is fundamental for discovering the role of ImP on glucose tolerance and insulin signaling. These researchers found that the action of the diabetes drug metformin is inhibited by ImP through complicated mechanisms involving various serine and threonine phosphorylations. Metformin performs its anti‐glycaemic activity by activating adenosine monophosphate‐activated protein kinase (AMPK) in the liver, inducing its phosphorylation at the threonine‐172 residue. On the other hand, ImP induces the phosphorylation of either serine‐485 or serine‐491, thereby attenuating the phosphorylation of AMPK at threonine‐172 and thus rendering the enzyme inactive. Moreover, the same research group has found that the inhibition of AMPK threonine‐172 phosphorylation happens via an upstream activity of ImP. ImP induces a phosphorylation on p38 (p38γ isoform) that induces a direct further phosphorylation of Akt serine–threonine kinase in the presence of ImP. Phosphorylated Akt induces the serine‐485 and serine‐491 phosphorylations on the AMPK enzyme that further block the threonine‐172 phosphorylation induced by metformin [82].

The same group of researchers continued their investigations on ImP‐related diabetes activity by showing that ImP, by activating p38γ, promotes p62 phosphorylation and subsequently activates the mechanistic target of rapamycin complex 1 (mTORC1) [82]. This phosphorylation cascade creates dysfunctional insulin signaling that involves the insulin receptors and a reduced quantity of insulin receptor substrate (IRS) proteins [83]. mTORC1 activation by ImP, again through a series of phosphorylations, blocks insulin signaling, leading to the degradation of IRSs; thus, Akt does not become activated. This is a serious dysregulation in insulin signaling, as the activated Akt pathway is needed to maintain normal metabolism and prevent both obesity and the development of type 2 diabetes [84].

Levels of ImP in the blood are statistically significantly higher in association with both diabetes and obesity, as well as inflammatory bowel disease [84]. Serum ImP levels were also positively associated with serum markers of inflammation. Diabetes and obesity are also associated with dysbiosis in the gut, with overrepresentation of species of the *Bacteroides* genus, underrepresentation of *Faecalibacterium prausnitzii* , and a general lack of diversity [85, 86]. Molinari et al. suggested that ImP overexpression is linked not to dietary histidine intake, but rather to an unhealthy diet with reduced intake of unsaturated fatty acids and fiber (a precursor to SCFAs) [85]. *F prausnitzii* is known for its ability to ferment fiber to produce SCFAs, particularly butyrate, which is low in deuterium due to hydrogen recycling [13]. There is no correlation between the amount of dietary histidine and ImP levels, indicating that the main factor controlling its levels is the gut microbiome. It can be inferred that insufficient supply of butyrate to the colon stresses the colonocytes, perhaps causing excess deuterium in the mitochondrial intermembrane space, and inducing oxidative stress as a consequence. ImP may ameliorate the situation through its ability to trap deuterium.

It is conceivable that ImP suppression of insulin receptors has a positive benefit for the brain, under conditions of systemic deuterium overload. Insulin resistance mostly affects muscle cells and fat cells. It induces elevated levels of blood sugar, which could be beneficial for assuring adequate glucose supply to the brain, particularly if the mitochondria are compromised by excessive deuterium. Indeed, it has been shown experimentally that brain glucose uptake upon insulin stimulation is increased in association with insulin resistance [87]. The lactate shuttle is a process by which astrocytes take up glucose, convert it to lactate, and deliver it to neurons [88]. Lactate is a low‐deuterium nutrient, compared to glucose [13]. High serum glucose enhances glycolysis in astrocytes and increases lactate production, as well as expression of the protein Monocarboxylate transporter 1 (MCT1), which transports lactate to the extracellular space [89]. This could be another beneficial outcome for the brain, at least in early stages of insulin resistance.

ImP cannot solely be regarded as a disease‐associated metabolite. During anti‐cancer radiation therapy, there can be a decrease in histidine levels in the intestine due to the knockdown of the microbiome. Relevant studies have shown that, during local radiation therapy for chest cancer (when histidine is diminished in the feces), oral administration of L‐histidine improved respiratory and heart function and retarded pathologically induced tissue damage in the heart and lungs due to radiation. However, the administration of L‐histidine could not provide radioprotection when there was a complete deletion of the microbiome in animals. Moreover, L‐histidine helped to maintain the normal microbiome bacterial populations in the gut. The protective and even therapeutic functions of L‐histidine in these cases seem to be due to the presence of ImP. During L‐histidine replenishment, ImP accumulated in the lungs and peripheral blood and was presumed to be the protective agent against the radiation‐induced lung and heart damage [90]. Furthermore, in the same study, ImP was shown to inhibit pyroptosis, an apoptosis‐like pro‐inflammatory cell death, in irradiated lung cells [91].

Many lessons can be learned from these cell studies. ImP acted as a pro‐inflammatory inhibitor, decreasing the levels of NF‐κB, inhibiting the activation of caspase‐1, caspase‐4, and caspase‐5 in the irradiated cells, and lowering the levels of IL‐1 and IL‐18. Concurrently, ImP elevated the levels of F‐actin and thus, by maintaining the cytoskeleton of cells, maintained the proliferation capacity and avoided cell death by pyroptosis. This study supports the importance of microbiome integrity to provide an ImP‐histidine‐related cytoprotective action against cardiopulmonary tissue destruction during cancer radiation therapies.

The same histidine metabolite from the gut microbiome can exert anti‐inflammatory activity, lower ROS, and perform anti‐glycaemic activity to treat inflammatory conditions like atopic dermatitis through the inhibition of the TNF‐α/IFN‐γ/IL4 axis [92]. However, ImP can also negatively affect glomerular filtration in patients with diabetic nephropathies to lead to renal failure [93]. Considering the studies described above, questions are raised as to how human health can be affected by the gut microbiome ImP‐producing bacterial populations. What might be some underlying factors that could induce the intestinal microbes to produce increased amounts of ImP, which in turn could lead to type 2 diabetes and obesity, two conditions that flourish in the western world? [94].

Through statistical analysis of multiple fecal metagenomes, researchers have identified three distinct “enterotypes” as characteristic clusters in the human microbiome: (1) *Bacteroides*, (2) *Prevotella*, and (3) *Ruminococcus* [95]. Of particular interest to us is the *Bacteroides* enterotype, where Bacteroides species dominate in the gut. This enterotype is associated with insulin resistance, decreased microbial diversity, and an increased risk of obesity and non‐alcoholic steatohepatitis (NASH) [96]. Interestingly, multiple studies have shown that repeated antibiotic treatment leads to the *Bacteroides* enterotype [97, 98]. Children suffering from watery diarrhea who were treated with multiple rounds of antibiotics showed an overabundance of Bacteroides species in the gut [97]. The popular antibiotics, fluoroquinolones and b‐lactams, resulted in a decrease in microbial diversity by 25%, associated with a statistically significant increase in the ratio of Bacteroidetes phylum to Firmicutes phylum (*p* = 0.0007). Furthermore, several unknown taxa belonging to the *Bacteroides* genus appeared following treatment with either antibiotic [98].

These results have potentially ominous implications for human health due to the overwhelming use of broad‐spectrum antibiotics in humans and in animals whose products are consumed by humans (e.g., dairy products) [99]. Type 2 diabetes patients are more susceptible to antimicrobial‐resistant infections and therefore would be prone to colonization of antibiotic‐resistant strains in their digestive tracts [100]. Furthermore, the consumption of antibiotic‐treated animal meat and products (e.g., dairy products), and the passage of the antibiotics to humans through diet are expected to further promote obesity and type 2 diabetes. High ImP production in the digestive tract and circulation of people who are exposed to broad‐spectrum antibiotics through their diet would be expected to create profound pathological changes in their microbiome over time.

## 9 Conclusion

The trillions of bacterial cells making up the human intestinal microbiome have been the subject of intensive research over the past decade. Hundreds of unique functional roles for the microbiome have been identified, and every major organ in the body has been characterized as having a gut‐organ axis. We have described here yet another role of the microbiome, one that is operating in parallel with every other function described to date, a role that could be considered fundamental and foundational to all others: that is, the continuous regulation of systemic deuterium and production of deupleted organic molecules, which is a necessary and universal prerequisite for efficient cellular function.

The microbiome can be thought of as an atomic sieve, ideally positioned near the entry point in the body for deuterium‐containing food and water. We have described a set of enzymes contained within the bacteria of the microbiome which act, via their high KIE with respect to deuterium, to “prune” hydrogens from incoming nutrients and shuttle them into the myriad functional roles they play within all cells throughout the body. In the process, deuterons are left behind, concentrated within species of bacteria adapted to that function. To facilitate deuterium excretion and prevent overaccumulation, we have also described here several classes of organic molecules, including carotenoids, PUFAs, and the amino acid histidine, all of which appear to play pivotal roles in capturing deuterons and attaching them to labile carbon atoms, thereby allowing for their excretion via the bowel and kidneys.

In addition to food and water, another source of deuterium‐containing organic molecules passing through the symbiotic sieve of the microbiome is heme and its breakdown products coming from the spleen via the liver and gallbladder. A set of microbial enzymes involved in heme breakdown can potentially scrub these breakdown products of deuterium, while capturing deuterium specifically at bis‐allylic carbon atoms and excreting it through the feces. All the while, they also generate deuterium‐depleted water.

**FIGURE 4:**
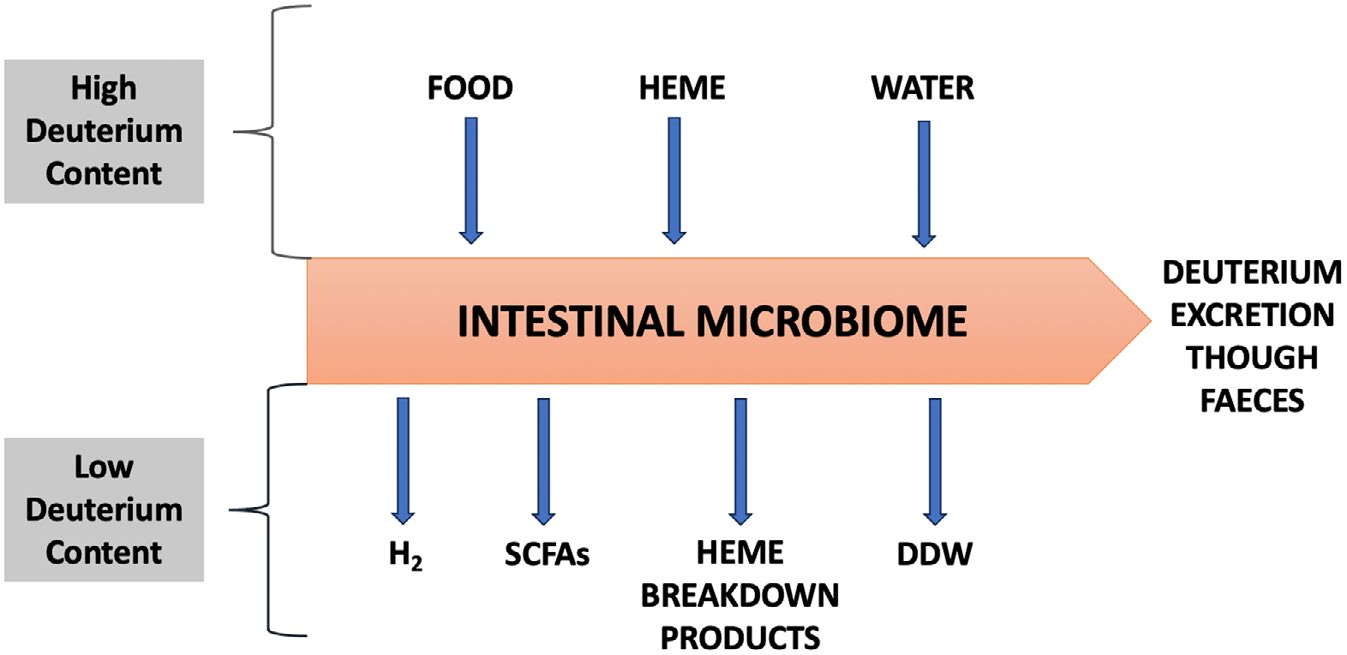
Schematic of the processes by which the microbiome facilitates delivery of deupleted protons to the mitochondria of the host cells.

This atomic sieve function of the microbiome, as schematized in Figure 4, is critical for maintaining efficient cellular functioning throughout the body, due to the vital importance of both deuterium depleted water (DDW) and deupleted organic molecules being in constant demand by every cell. The example of histidine/histamine metabolism and its downstream product ImP can illustrate how excess deuterium involved in this metabolic pathway leads to cellular dysfunction and consequently systemic pathologies.

Several bacterial and endogenous enzymes and molecules continuously act to keep this sieve function of the microbiome operational and optimized. What is documented here may represent the tip of the iceberg with respect to deuterium regulation throughout the body. Repeated exposure to antibiotics may disrupt the delicate balance in the microbiome, leading to an impaired ability to protect the mitochondria from deuterium toxicity and resulting chronic disease. A greater understanding of the role the microbiome is playing to maintain systemic deuterium sequestering and homeostasis creates a new lens through which to view each gut‐organ axis. It clarifies both the physiological role and health benefits of a low‐deuterium diet.

## Author Contributions

S.S. wrote the first draft. S.S., A.M.K., and G.N. contributed to many rounds of expansion and revision.

## Conflicts of Interest

The authors declare no conflicts of interest.

## Data Availability

The authors have nothing to report.
